# Supplementary material for: I Wanna Draw Like You: Inter- and Intra-Individual Differences in Orang-Utan Drawings
Source: Animals (Basel). 2021 Nov 9;11(11):3202. doi: 10.3390/ani11113202 (PMC8614565; doi:10.3390/ani11113202)
Supplement: Supplementary file 1 [file animals-11-03202-s001.zip › animals-1400892-supplementary.pdf]

Supplementary material for Interindividual and intraindividual differences in orangutans drawings

Marie Pelé[1], Gwendoline Thomas[2], Alaïs Liénard[2], Masaki Shimada, Cédric Sueur[4,5]
1: Anthropo-Lab, ETHICS EA7446, Lille Catholic University, Lille, France
2 : Université Sorbonne Paris Nord - UFR LLSHS, Paris, France
3 : Department of Animal Sciences, Teikyo, University of Science, Uenohara, Yamanashi, Japan
4: Université de Strasbourg, CNRS, IPHC UMR 7178, Strasbourg, France
4 : Institut Universitaire de France, Paris, France

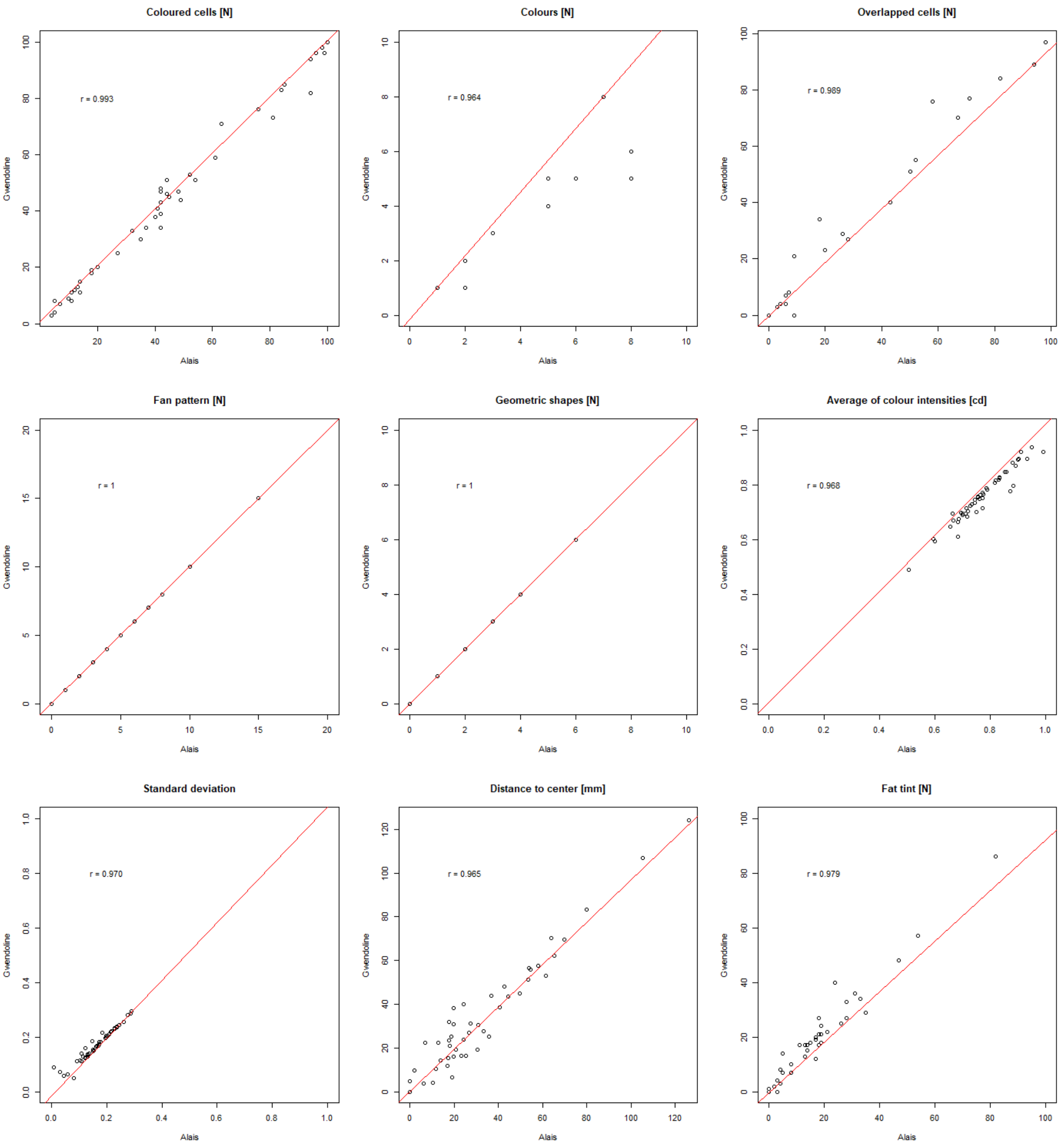

Figure S1. Inter-observer correlations for quantitative variables in the classical analysis.

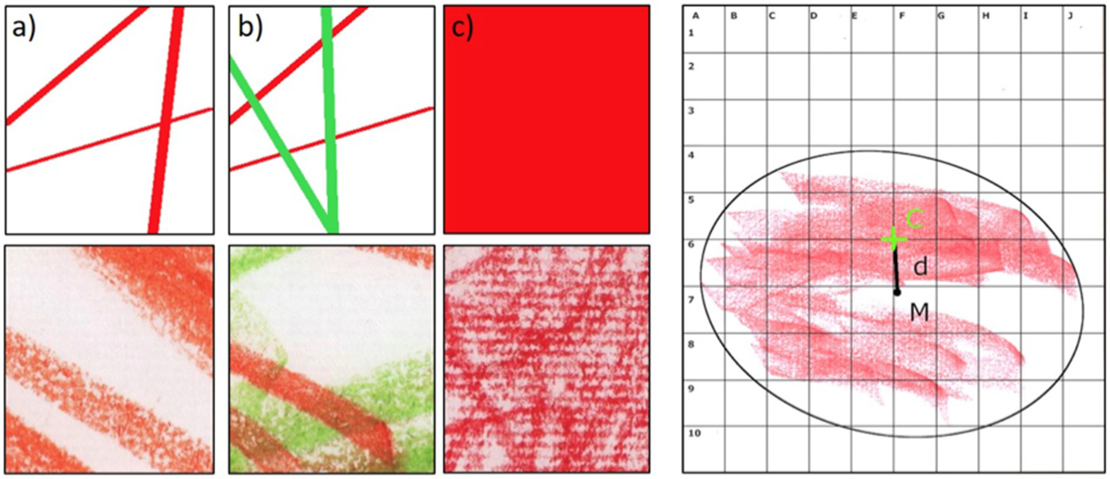

Figure S2: On the left, schematic (top) and actual (bottom) examples of a) a covered cell, b) an overlapped cell and c) solid colour rate. On the right, calculation of the distance to the centre. C is the centre of the paper sheet, M is the centre of the drawing ellipse, and d is the diameter.

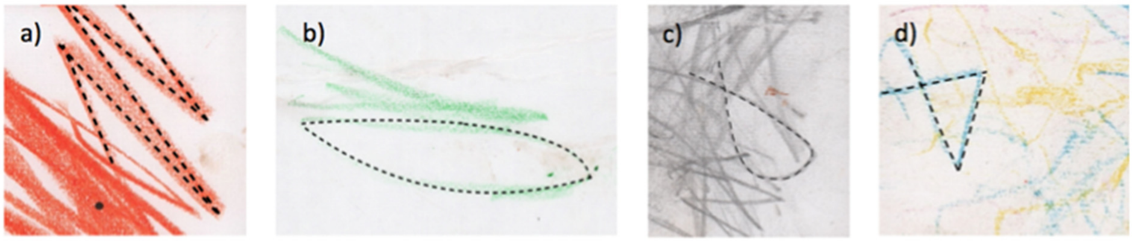

Figure S3: examples of a) a fan pattern, b) a circle, c) a loop and d) a triangle.

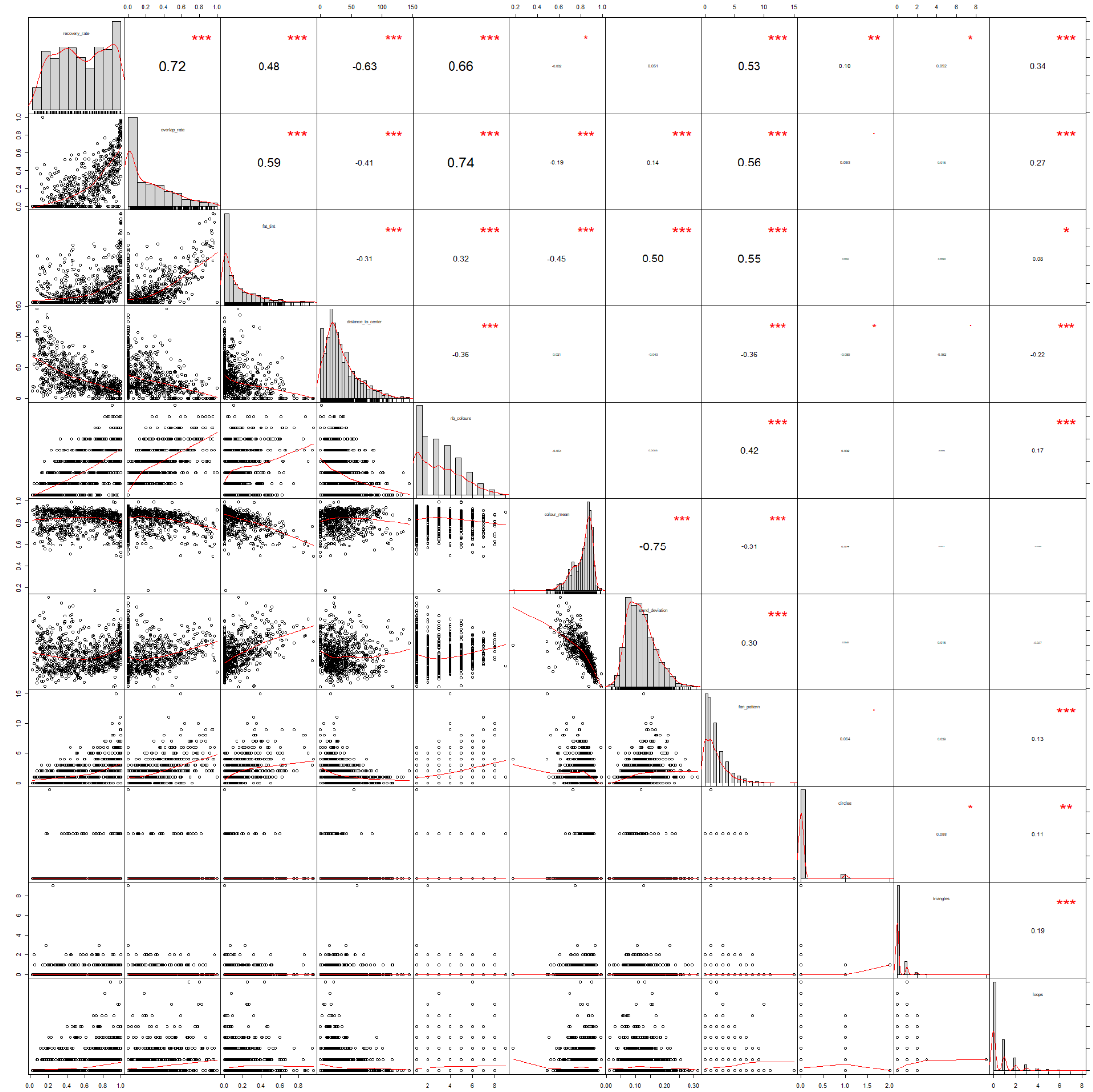

Figure S4. Correlation chart of our quantitative variables. *:p<0.05, **p<0.01,***:p<0.001. The number indicates the correlation.

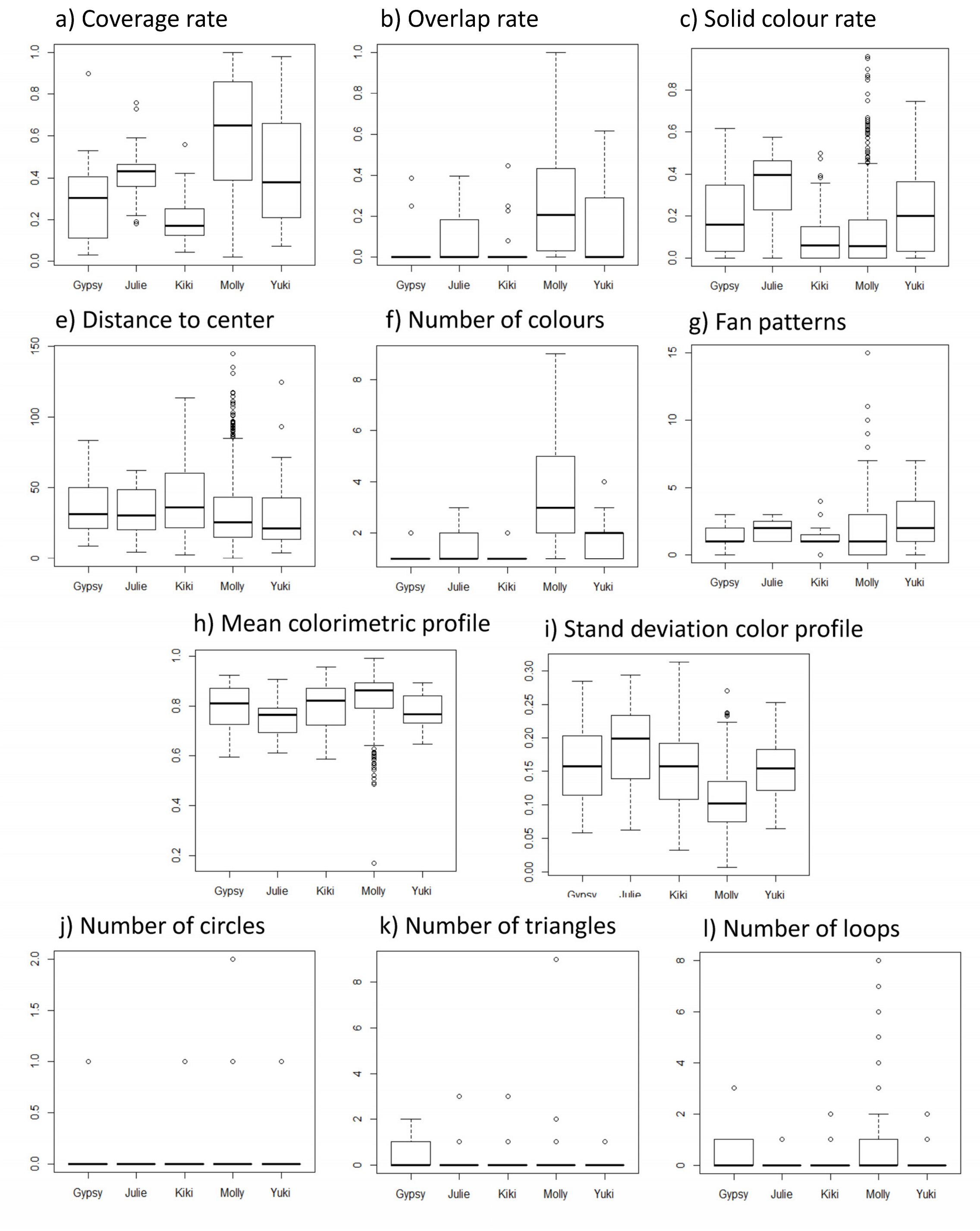

Figure S5: Boxplots of each drawing metric per individual

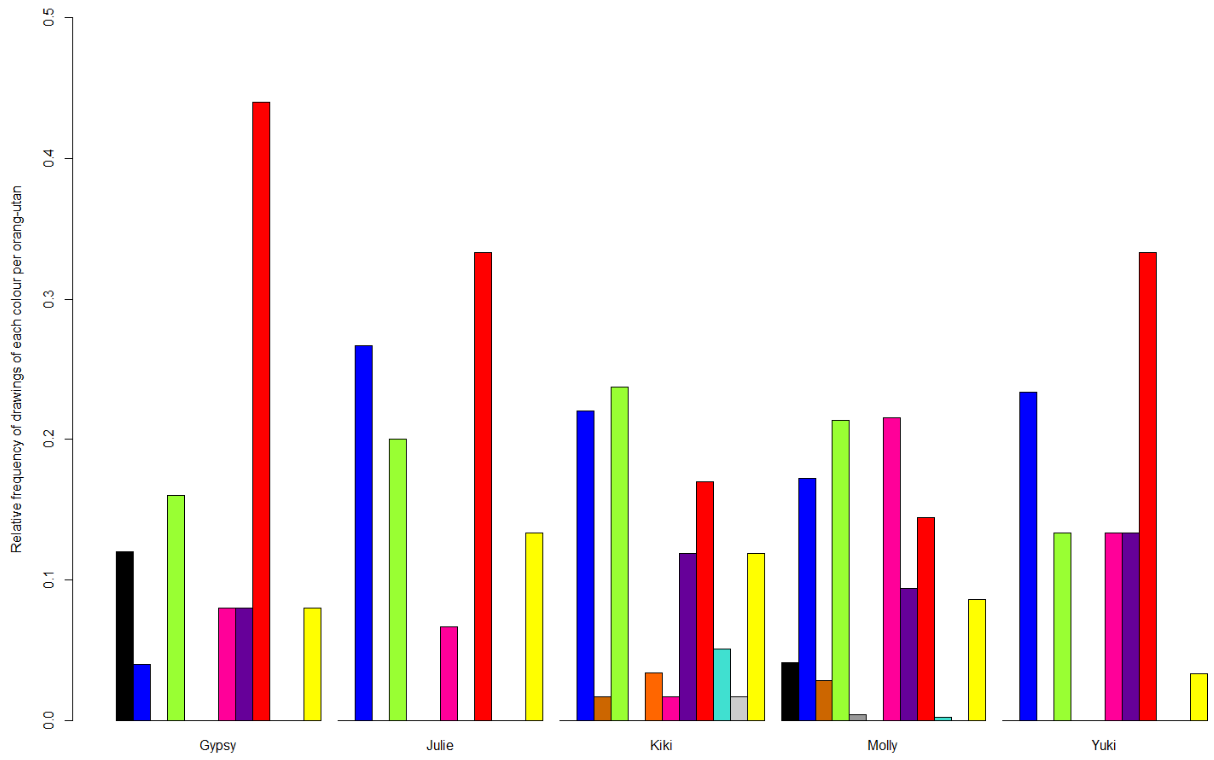

Figure S6. Frequency of drawings per main colour per individual.

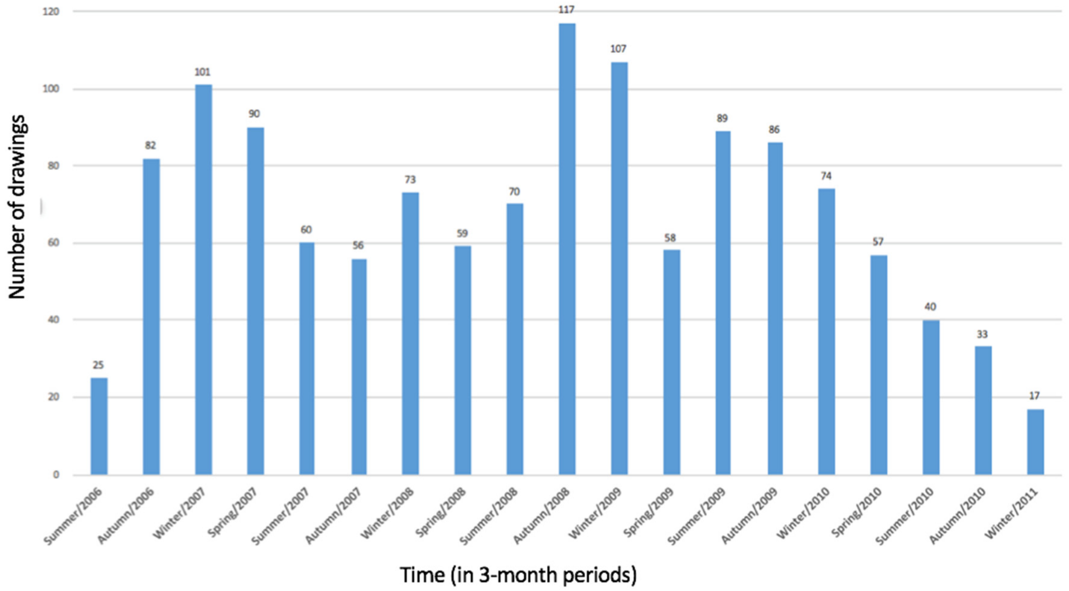

Figure S7: Number of drawings according to the seasons over the years. The exact number is specified above the bars.

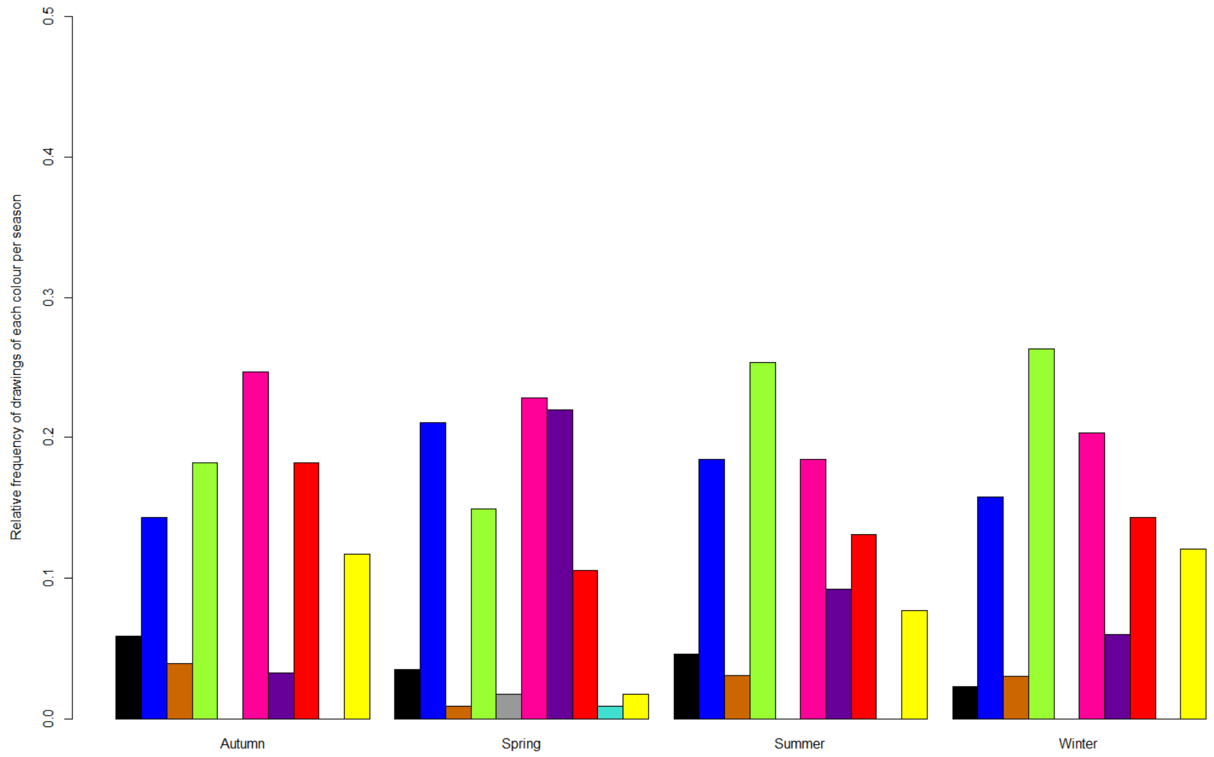

Figure S8. Frequency of Molly's drawings per main colour per season.

Table S1: pairwise comparisons tests (p-values) for each dimension between the five individuals

**Dim1**	**Gypsy**	**Julie**	**Kiki**	**Molly**
**Julie**	0.1844	-	-	-
**Kiki**	0.3149	0.0152	-	-
**Molly**	0.016	0.9006	3.60E-09	-
**Yuki**	0.1525	0.9006	0.0025	0.8234
**Dim2**	Gypsy	Julie	Kiki	Molly
**Julie**	0.081	-	-	-
**Kiki**	0.932	0.045	-	-
**Molly**	4.80E-11	8.30E-14	< 2e-16	-
**Yuki**	0.907	0.045	0.907	5.00E-12
**Dim3**	Gypsy	Julie	Kiki	Molly
**Julie**	0.643	-	-	-
**Kiki**	0.301	0.643	-	-
**Molly**	0.041	0.301	0.301	-
**Yuki**	0.301	0.601	0.643	0.643

Table S2: pairwise comparisons tests (p-values) for each metric of Dimension 1

**Coverage rate**	**Gypsy**	**Julie**	**Kiki**	**Molly**
**Julie**	0.13059	-	-	-
**Kiki**	0.17047	**0.00479**	-	-
**Molly**	**1.50E-08**	**0.01076**	**< 2e-16**	-
**Yuki**	0.07067	0.99024	**0.00029**	**0.0004**
**Overlap rate**	Gypsy	Julie	Kiki	Molly
**Julie**	0.482	-	-	-
**Kiki**	0.869	0.372	-	-
**Molly**	**8.40E-06**	**0.018**	**5.60E-13**	-
**Yuki**	0.112	0.549	**0.032**	**0.018**
**Number of colours**	Gypsy	Julie	Kiki	Molly
**Julie**	0.541	-	-	-
**Kiki**	0.931	0.541	-	-
**Molly**	**1.30E-10**	**1.90E-05**	**< 2e-16**	-
**Yuki**	0.146	0.541	0.093	**2.10E-06**
**Fan patterns**	Gypsy	Julie	Kiki	Molly
**Julie**	0.5247	-	-	-
**Kiki**	0.8489	0.346	-	-
**Molly**	0.329	0.9918	**0.0406**	-
**Yuki**	**0.0406**	0.3048	**0.0044**	**0.0406**
**Distance to centre**	Gypsy	Julie	Kiki	Molly
**Julie**	0.911	-	-	-
**Kiki**	0.714	0.668	-	-
**Molly**	0.714	0.912	**0.035**	-
**Yuki**	0.714	0.912	0.271	0.912
**Solid colour rate**	Gypsy	Julie	Kiki	Molly
**Julie**	0.0212	-	-	-
**Kiki**	0.0189	**3.50E-06**	-	-
**Molly**	**0.0287**	**3.50E-06**	0.319	-
**Yuki**	0.5568	**0.0461**	**0.0015**	**0.0015**

Table S3: pairwise comparisons tests (p-values) for each metric of Dimension 2

**Colour mean**	**Gypsy**	**Julie**	**Kiki**	**Molly**
**Julie**	0.2651	-	-	-
**Kiki**	0.8763	0.2651	-	-
**Molly**	0.1266	0.0057	0.0057	-
**Yuki**	0.5863	0.5263	0.5863	0.0059
**Standard deviation of mean colour**	Gypsy	Julie	Kiki	Molly
**Julie**	0.0335	-	-	-
**Kiki**	0.5064	0.0022	-	-

**Molly**	1.40E-07	1.20E-11	5.60E-11	-
**Yuki**	0.7355	0.0135	0.6962	9.40E-08

Table S4: pairwise comparisons tests (p-values) for each metric of Dimension 3

Circles	Gypsy	Julie	Kiki	Molly
**Julie**	0.88	-	-	-
**Kiki**	0.88	0.88	-	-
**Molly**	0.88	0.88	0.88	-
**Yuki**	0.89	0.88	0.88	0.88
**Triangles**	Gypsy	Julie	Kiki	Molly
**Julie**	1	-	-	-
**Kiki**	0.45	0.45	-	-
**Molly**	0.45	0.54	0.45	-
**Yuki**	0.45	0.45	0.89	0.65
**Loops**	Gypsy	Julie	Kiki	Molly
**Julie**	0.5006	-	-	-
**Kiki**	0.5006	0.835	-	-
**Molly**	0.5006	0.1012	**0.0022**	-
**Yuki**	0.5542	0.835	0.835	0.079

Table S5: Loadings of the metrics on the three PCA dimensions of our dataset for Molly. Bold values indicate the dimension in which each variable is retained.

	Dim.1	Dim.2	Dim.3
**coverage rate**	**0.83259466**	0.35898791	-0.03754139
**Overlap rate**	**0.86645016**	0.11386246	-0.15141994
**Solid colour rate**	**0.82093692**	-0.20890006	-0.06820954
**distance to centre**	**-0.59224515**	-0.4847952	0.0366672
**Number of colours**	**0.70657478**	0.21081542	-0.29585089
**Colour mean**	-0.5250608	**0.71138628**	-0.22231713
**Standard deviation of mean colour**	0.59217275	**-0.65153752**	0.21292017
**fan pattern**	**0.75009581**	-0.06012067	-0.05905462
**circles**	0.09579288	0.18189447	**0.50532385**
**triangles**	0.07651598	0.16002544	**0.70546716**
**loops**	0.30038742	0.37499276	**0.49370317**
